# Research on the Impact of Government Environmental Information Disclosure on Green Total Factor Productivity: Empirical Experience from Chinese Province

**DOI:** 10.3390/ijerph19020729

**Published:** 2022-01-10

**Authors:** Liang Zhao, Liangyu Chen

**Affiliations:** 1 Tourism School, Hubei University, Wuhan 430062, China; zhaoliang@hubu.edu.cn; 2 School of Tourism and Hospitality Management, Wuhan City Polytechnic, Wuhan 430064, China

**Keywords:** environmental supervision, environmental information disclosure, green economy, threshold effect

## Abstract

Government environmental information disclosure is an important means to promote environmental supervision and law enforcement, and improve the level of environmental management. In order to explore the impact of government environmental information disclosure on the sustainability of urban economic growth, this paper uses the Pollution Information Transparency Index (PITI) to measure the degree of government environmental information disclosure, studies its effect on green total factor productivity through two-way fixed effect model and systematic GMM estimation method, and further adopts threshold model to study whether there is heterogeneity in this effect. The results show that: (1) Each unit of government environmental information disclosure will increase green total factor productivity by 0.2 units. (2) Considering the endogeneity, the promotion of government environmental information disclosure to green total factor productivity has increased. (3) The degree of government environmental information disclosure plays a non-linear role in the path of green total factor productivity. The greater the degree of economic development, the more obvious the effect of government environmental information disclosure on green total factor productivity. Therefore, this paper believes that the government should strengthen the disclosure of environmental information based on the urban economic development to ensure the sustainability of urban economic development.

## 1. Introduction and Literature Review

Environmental information disclosure is divided into corporate environmental information disclosure and government environmental information disclosure. The former refers to the disclosure of information related to the environmental impact of its business activities and corporate environmental behavior, and the latter refers to the information that the government will learn when it performs environmental protection duties [1,2]. Disclosure needs to be made so that the public knows. On the one hand, this is the protection of the public’s right to know [3]; on the other hand, it can also be used as an administrative management method to increase restraint on polluting enterprises [4]. The disclosure of pollution source information refers to the public disclosure of information such as the location of the source of pollution that has caused or may cause environmental pollution, the types of pollutants, and the number of pollutants discharged, so that the public can know. In 1986, the promulgation of the “Emergency Planning and Community Right-to-Know Act” in the United States marked the initial birth of this administrative management system, which required disclosure of potential pollution source information to the public and the government. If the discharge of a certain pollutant substance exceeds a certain threshold, it is also necessary to report the pollutant list. Since then, the system of “environmental disclosure” has gradually been valued by all countries.

China’s “Environmental Protection Law of the People’s Republic of China” was promulgated in 1989. Later, in 2007, the 17th National Congress of the People’s Republic of China first clarified the goal of “ecological civilization construction”. In the same year, the “Environmental Information Disclosure Measures (Trial)” was passed and formally implemented in 2008. The birth of the measures was systematically stipulated in procedures. The method of public disclosure of environmental information in China has created a legislative precedent in the administrative system of public disclosure of environmental information in my country. On 1 January 2015, the newly revised Environmental Protection Law also came into effect.

Existing studies have proved that a sound environmental pollution information disclosure system has many benefits: Yu found that environmental information disclosure has a positive impact on economic performance, and companies that fully disclose their environmental information have better economic performance [5]; Shi Beibei Evans found that environmental disclosure and the structure of foreign direct investment are significantly related. A strict environmental disclosure system can increase the proportion of investment in clean companies [6]; Evans believes that environmental information disclosure can reduce social costs [7]; some scholars from the perspective of the system, explain the significance of environmental disclosure to democracy and the public’s right to know [8]. Correspondingly, some studies have also found that environmental pollution information disclosure also has certain drawbacks: Cohen et al. found that environmental disclosure will increase social costs [9]. This article believes that China is now in a new period of in-depth promotion of the battle against environmental pollution, and environmental information disclosure is an important tool to control environmental pollution, so the advantages of strengthening environmental information disclosure obviously outweigh the disadvantages at this stage.

At present, there are many studies on the factors affecting environmental pollution: Lu Ming et al. found that population gatherings reduce the level of environmental pollution per unit area [10]. He et al. believe that environmental pollution is related to three dimensions: distance, density, and degree of division [11]. Copeland believes that the transfer of technology level in international trade is conducive to the improvement of environmental problems in developing countries, and puts forward the view that the improvement of technology level will affect environmental conditions [12]. Huang Maoxing and others analyzed the relationship between environmental pollution, economic development and environmental management, and demonstrated that economic development requires excellent environmental governance capabilities [13]. Ke et al. (2021) studied the spatial effect of urban innovation performance on the ecological footprint, and concluded that urban innovation efficiency can significantly inhibit the ecological footprint of the region and surrounding areas [14]. Fan et al. indicates that there is a U-shaped curve relationship between environmental regulation and green innovation efficiency [15]. Wang et al. pointed out that the emergence of urban morphology is related to ecological efficiency, and the maximum patch index and patch density can improve the urban environment [16].

Compared with environmental pollution, the research on green total factor productivity is more important. The growth of green total factor productivity is an important indicator of the quality of a country’s economic growth and technological progress. It also improved management efficiency, and it has become the core of modern economic growth. Whether it is traditional neoclassical economics or endogenous economic growth theory, on the one hand, it emphasizes that the measurement standard of economic growth is GDP and per capita income level; on the other hand, it emphasizes that the input elements of economic growth include capital, labor or human capital, as well as technological progress and systems, etc. However, as the ecological environment continues to deteriorate and environmental pollution continues to affect residents’ health and social welfare, GDP and per capita income are no longer the only yardsticks for measuring social progress. It is necessary to incorporate resource element input and environmental impact into the mainstream economic growth theory by utilizing the “Green Economic Growth Theory” [17]. Green economic growth, as the extension and development of sustainable development theory and a tool to achieve sustainable development, has become the development strategy of many countries. In 2011, the Asia-Pacific Economic Organization (OECD) defined green economic growth as: “Promote economic growth and development, while ensuring that natural resources continue to provide the resources and environmental services that our well-being depends on.” The United Nations Environment Program defines green economic growth as “an economy that improves human well-being and social equity while significantly reducing environmental risks and ecological scarcity.” There are three standards for green economic growth: First, the economy maintains a certain growth rate, ensures the stability of the macro economy, achieves full employment, controls inflation, and increases per capita national income; second, the ecosystem and environmental quality are improved to achieve environmental protection sustainability; third, to achieve inclusive and peaceful growth, to achieve fair and reasonable distribution and sharing between regions, urban and rural areas, and to achieve overall Pareto improvement. Traditional total factor productivity is the driving force for the sustained growth of the traditional economy, while green total factor productivity is the driving force for achieving green economic growth [18], so it is necessary to accelerate technological and institutional innovation to continuously achieve green total factor productivity growth.

The measurement of total factor productivity is troubled by factors such as the variability of input and output factor selection and measurement uncertainty. Each measurement method has a certain degree of advantages and disadvantages, which affect the accuracy of the result of total productivity. In addition, according to the definition of total factor productivity, it can be seen that the quantity and quality of labor, capital level and distribution pattern, total energy consumption and efficiency level, and other original input factors all have an impact on total factor productivity; total factor productivity is used as input and output as a measure of efficiency, the technological level of a certain region or industry can affect the conversion efficiency of input and output. Resource allocation efficiency, technical knowledge stock, technological advancement, and intermediary service capabilities all have an impact on this conversion efficiency. Therefore, the impact that affects the level of total factor productivity includes not only a single production factor link such as labor and capital, but also a series of soft environmental constraints such as innovation atmosphere, technological level, collaboration ability, and social capital.

Compared with traditional total factor productivity, including energy consumption, environmental pollution and other factors into the analysis framework to investigate green total factor productivity has become a choice in the context of tightening constraints on energy consumption and environmental pollution. Based on the existing research on the factors affecting total factor productivity, the existing factors affecting green production efficiency are mostly concentrated on the types of environmental regulations, the degree of technological development that affects energy efficiency, and market competition forces companies to upgrade their technology according to the research framework of productivity influencing factors [19]. Existing research on (green) total factor productivity influencing factors is carried out from different levels, involving the scale of production factors, technical level, ownership form, environmental regulation intensity, aging, policy, industry etc., [20,21,22,23], forming a relatively complete total factor with an analysis framework of productivity influencing factors [24,25]. However, the existing research species of green total factor productivity pays more attention to factors that affect energy efficiency and environmental pollution such as environmental regulations and technical levels, while traditional total factor productivity pays more attention to the scale input of production factors and the improvement of resource allocation efficiency. In knowledge spillovers and other factors, there is a big difference in their attention to influencing factors. The main problem in the existing research on the influencing factors of (green) total factor productivity is: assuming that differences in different industries or regions have no difference in the impact of (green) total factor productivity, and the size and scope of the impact are consistent. It does not distinguish between the different effects of industry and region heterogeneity on total factor productivity, and adopts a “one size fits all” approach, which is likely to cause deviations in the estimation results.

For the research on government environmental information disclosure and environmental supervision level, Oates takes government revenue and expenditure as the entry point. The basic assumption is that government expenditure on environmental supervision is directly proportional to the level of environmental supervision. Individual taxation, while government expenditure lies in environmental supervision, infrastructure construction, etc. Therefore, if the government in a certain area is keen to reduce taxes and increase infrastructure investment, it will reduce the government’s fiscal revenue from investing in environmental supervision, which will affect environmental supervision [26]. Using game theory as a tool, Wang et al. started with the relationship between government and enterprise, and concluded that the dynamic game between government and enterprise is an important factor affecting environmental supervision [27]. Through empirical research, Zheng et al. concluded that public participation is helpful to the government’s environmental supervision and therefore beneficial to local environmental protection. The more the public pays attention to environmental issues, the sooner the city can enter the inflection point of the Kuznets curve [28].

In summary, environmental information disclosure is an important means for the government to control environmental pollution, and green total factor productivity is a key index to evaluate the sustainability of urban economic growth, but there are still few studies that focus on the relationship between government environmental information disclosure and green total factor productivity, or that which discuss the impact of government environmental information disclosure on green total factor productivity. In order to explore whether government environmental information disclosure can promote the sustainability of urban economic growth, this paper uses China’s provincial-level data from 2010 to 2018, selects the slack-based directional distance SBM function proposed by Fukuyama and Weber (2009), and the Luenberger productivity index with additive structure proposed by Chambers et al. (1996) to calculate the green total factor productivity, on this basis, then studies the impact of government environmental information disclosure on green total factor productivity based on two-way fixed effect model, solves the endogenous problem through system GMM estimation, and further adopts threshold model to explore its impact and whether there is heterogeneity in different regions

The marginal contributions of this article are as follows: (1) The impact of regional government environmental information disclosure on green total factor productivity is studied, and it provides empirical evidence for whether government departments should continue to strengthen environmental information disclosure. (2) It measures the level of green total factor productivity and government environmental information disclosure at the provincial level in China, and provides reliable data for future research. (3) It studies the difference of green total factor productivity in different types of cities affected by government environmental information disclosure, and provides decision-making suggestions for which attributes of cities should increase environmental information disclosure.

## 2. Green Total Factor Productivity Measurement Method

The current measurement methods of total factor productivity mainly include Solow Residual Value Method, Cobb Douglas Production Function Method, and Production Frontier Method. The measurement of the rate of change of total factor productivity can be roughly divided into two types: parametric and non-parametric methods. Among them, the parameter method is based on the production function, and obtains the rate of change of total factor productivity by calculating the residual value of production. When using the parameter method, the first problem is to determine the production function, which will directly affect the final result. The non-parametric method analyzes production efficiency from different angles. It avoids the problem of production function and directly measures changes in total factor productivity from input and output. The green total factor productivity is measured on the basis of total factor productivity, and calculated after introducing environmental factors.

This paper selects the SBM function based on the slack directional distance proposed by Fukuyama and Weber [29] and the Luenberger productivity index with additive structure proposed by Chambers et al. [30] to calculate the green total factor productivity. Inspired by the idea of global ML index proposed by [31], the global directional distance function and global Luenberger index based on SBM are constructed. The global production frontier is constructed after detecting the production technology in the entire time period, which to a certain extent avoids “technical retrogression” and the phenomenon of unsolvable linear programming that may occur when using mixed directional distance functions. The global Luenberger index constructed on this basis is cyclically cumulative. It can not only analyze short-term changes in green total factor productivity, but also observe its long-term trends. The specific form of the SBM directional distance function is as follows:

(1) $$S^{G,k^{\prime }}\left(x^{t,k^{\prime }},y^{t,k^{\prime }},b^{t,k^{\prime }},g^{x},g^{y},g^{b}\right)=\begin{array}{c} max\\ S_{x},S_{y},S_{b} \end{array}\frac{1}{2N}\overset{N}{\underset{n=1}{\sum }}\frac{S_{n,x}^{G,k^{\prime }}}{x_{n}^{t,k^{\prime }}}+\frac{1}{M+1}\left[\overset{M}{\underset{m=1}{\sum }}\frac{S_{m,y}^{G,k^{\prime }}}{y_{m}^{t,k^{\prime }}}+\overset{I}{\underset{i=1}{\sum }}\frac{S_{i,b}^{G,k^{\prime }}}{b_{i}^{t,k^{\prime }}}\right]$$

(2) $$s.t.\overset{T}{\underset{t=1}{\sum }}\overset{K}{\underset{k=1}{\sum }}z^{t,k}x_{n}^{t,k}+S_{n,x}^{G,k^{\prime }}=x_{n}^{t,k^{\prime }};$$

(3) $$\overset{T}{\underset{t=1}{\sum }}\overset{K}{\underset{k=1}{\sum }}z^{t,k}y_{m}^{t,k}-S_{m,y}^{G,k^{\prime }}=y_{m}^{t,k^{\prime }};$$

(4) $$\overset{T}{\underset{t=1}{\sum }}\overset{K}{\underset{k=1}{\sum }}z^{t,k}b_{i}^{t,k}-S_{i,b}^{G,k^{\prime }}=b_{i}^{t,k^{\prime }}$$

(5) $$z^{t,k}\geq 0;S_{n,x}^{G,k^{\prime }}\geq 0;S_{m,y}^{G,k^{\prime }}\geq 0;S_{i,b}^{G,k^{\prime }}\geq 0;n=1\cdots N,m=1\cdots M,i=1\cdots I$$

Among them, $S^{G,k^{\prime }}$ represents the distance between the decision-making unit *k*^’ and the “global” production frontier, and the measured distance is actually specified as along $\;g=\left(-x^{t},y^{t},-b^{t}\right)$, reduce input as much as possible, increase expected output and reduce undesired output. $\left(x^{t,k^{\prime }},y^{t,k^{\prime }},b^{t,k^{\prime }}\right)$ is the input and output of the *k*’th provincial region, $\left(g^{x},g^{y},g^{b}\right)$ is the direction vector of the ***k*** provincial region, which represents the decrease of input, the increase of expected output or the decrease of undesired output.$\;S_{n,x}^{G,k^{\prime }},S_{m,y}^{G,k^{\prime }},S_{i,b}^{G,k^{\prime }}$ respectively represent the ***n*** element input, the *m*-th expected output, and the slack vector of the ***i*** undesired output. On this basis, the global Luenberger index GL, which measures technological inefficiency, can be expressed as: ${\mathrm{GL}}_{t}^{t+1}=S^{G,k^{\prime }}\left(t+1\right)-S^{G,k^{\prime }}\left(t\right)$.

The data envelopment analysis model is not only applicable to the calculation of total factor productivity, but also widely used to calculate different types of efficiency values such as innovation efficiency [32,33,34], ecological efficiency [35], coupling efficiency, and economic efficiency [36,37,38]; its production frontier of the data envelopment analysis model is composed of piecewise linear functions. The piecewise linear functions will be parallel to the coordinate axis in the space coordinate system. This is the source of slack variables. This article takes a single input and a single output as an example. In the CRS model, the frontier of the single input and single output is a ray starting from the center of the coordinate axis. The slack variable in the single-output CRS radial model is definitely 0, that is, there is no slack problem. 

The significance of the relaxation vector is that when each element of the relaxation vector is 0, its observation point is the best point, and there is no technical inefficiency. On the contrary, there is room for improvement. When $S_{n,x}^{G,k^{\prime }},\;S_{m,y}^{G,k^{\prime }},\;S_{i,b}^{G,k^{\prime }}$ are all greater than 0, this indicates that the actual expected output is less than the expected output of the frontier boundary. Whether the distance of the observation point is optimal can be measured by the relaxation vector; if the expected output is insufficient or the undesired output is too much, and the redundancy is greater, it will lead to $S_{n,x}^{G,k^{\prime }},\;S_{m,y}^{G,k^{\prime }},\;S_{i,b}^{G,k^{\prime }}$ becoming larger.

In 1997, Chung et al. extended it to a directional distance function ML index [39] that can measure environmental factors, which can solve the problem of evaluating undesired output.. However, whether it is the M index or the ML index, both of these indexes need to choose the measurement angle under the assumption of cost minimization or profit maximization, that is, an input-based measurement method or an output-based measurement method. The Luenberger productivity index does not need to choose the measurement angle, and can simultaneously consider the reduction of input and the increase of output, and the maximization of profit, corresponding to the hypothesis of profit maximization, and can also consider the case of minimizing costs and maximizing benefits. Therefore, the Luenberger productivity index is a generalization of the M and ML indexes [40]. This paper considers the environmental factors in the model, and uses the equation form as follows:

(6) $$\vec{D}\left(x,y,b;\;\vec{g}\right)=\;\sup\left\{\beta :\left(y,b\right)+\beta \vec{g\;}\in \;P\left(x\right)\right\}$$

In the above equation, x is the input variable, y is the expected output variable, and b is the undesired output variable. $\vec{g}$ = ($\vec{g_{x}},\;\vec{g_{y}},\;\vec{g_{b}})$ is the direction vector. This direction vector can be used to consider both the decrease in input and the increase in output. This article assumes that g=(y,−b); that given input index x, the required output and undesired output are proportionally expanded and reduced, and β is the non-deterministic output of the increase in y and the decrease in b. The maximum possible value of the expected output is when assuming that each decision-making unit uses N input variables, that is, $\;x=\left(x1,\ldots ,xN\right)\in \;R_{+}^{N}$ to obtain M kinds of expected outputs $y=\left(y1,\ldots ,yM\right)\in R_{+}^{M}$ and I kinds unexpected output, $b=\left(b1,\ldots ,bI\right)\in R_{+}^{I}$. Then, $P^{t}\left(x\right)$ represents the set of production possibilities within *t* = 1, …, T: $P^{t}\left(x\right)=\left\{\left(y^{t},b^{t}\right):x^{t}\to \left(y^{t},b^{t}\right)\right\},\;x\in \;R_{+}^{N}$.

In order to improve the comparability of the technical efficiency of the decision-making unit, Oh defines the production frontier technology as the union of all current production possibilities set, $P^{G}\left(x\right)=P^{1}\left(x^{1}\right)\cup P^{2}\left(x^{2}\right)\cup \ldots \cup P^{T}\left(x^{T}\right)$. Under a single production frontier, the calculated green total factor productivity can be compared between each decision-making unit and each time period.

In view of the DDF production function defined above and the production possibility set of the global production frontier, the global ML index obtained in this paper is cumulative and circular, avoiding the incomparable ML index. The GML index is defined as follows: where *t*, *t* + 1 represents the time period and ${\vec{D}}^{G}$ is the distance function defined on the global technology set.

(7) $$GML_{t}^{t+1}=\frac{1+{\vec{D}}^{G}\left(x^{t},y^{t},b^{t};y^{t},b^{t}\right)\;}{1+{\vec{D}}^{G}(x^{t+1},y^{t+1},b^{t+1};y^{t+1},b^{t+1}\;}$$

Previous studies usually decompose the change of total factor productivity into technology change ($TC_{t}^{t+1}$) and efficiency change $\left(EC_{t}^{t+1}\right)$ based on the principle of its measurement. Weimin’s [37] decomposition principle is measured by the sum of the two in this article. The reason is that, on the one hand, the green total factor productivity change rate measured in this article is a relative concept, and it is actually measured by changes in TFP. These changes may be due to the better combination of existing factors and production, not necessarily that it is related to the introduction of new production factors or technological innovation; on the other hand, changes in efficiency reflect the ability of provinces and regions to absorb existing knowledge, technology and production factors, and efficiency changes should be a key variable in economic development research. From these two perspectives, the overall productivity index integrates technical changes and efficiency changes in the usual sense, and it is more reasonable to embody green total factor productivity as the continuous process of each decision-making unit to catch up with the global frontier measure.

According to the above calculation method, the following table presents the calculation results of the overall green total factor productivity of the country and the three major regions of eastern, central, and western during the period 2010–2018. The data in the table shows that whether it is from the whole country as a whole, or from the perspectives of the eastern, central, and western regions, respectively, within the time frame of the calculation results, China’s green total factor productivity level has shown a trend of first rising, then falling, and then rising. Although in some years, there has been a significant decline in green total factor productivity. For example, from 2012 to 2013, the total factor productivity of the eastern, central, and western regions fell as high as −24.4%, −12.5%, and −15.2%, respectively. The overall decline in the country was as follows: −18.8%, but on the whole, China’s green total factor productivity’s average annual growth rate during 2006–2018 was about 1.9%. The national and regional productivity development over the years 2010–2018 has distinct phase characteristics: 2012 is clearly a turning point in the time series studied, and the green total factor productivity of the country as a whole and the eastern, central, and western regions in 2010–2011. During the year, there has been a continuous and stable increase. The increase rate reached the largest during 2011–2012. The growth rate of the whole country and the eastern region reached 13.9% and 21.8%, respectively, and the growth rate of the central and western regions was about 8%. In the time series studied, except for the eastern region, the total factor productivity of the whole country and the central and western regions reached the maximum in 2012, but this growth trend did not continue. In 2013, the total factor productivity level of each region was large. However, since 2014, the green total factor productivity of various regions has resumed its upward trend again. However, until 2018, except for the eastern region, the central and western regions and the entire country’s overall green total factor productivity have not recovered to the 2012 level. In terms of the differences between regions, Table 1 shows that the green total factor productivity of the central and western regions has been below the national average for a long time, while the eastern region is far ahead. There is a huge gap between regions. Generally speaking, from 2010 to 2018, China’s total factor productivity level showed the lowest level in the central region, and the western and eastern regions gradually increased.

There are many factors that contribute to the above differences, such as urbanization rate, geographic location, industrial structure, manpower and R&D capital, regional economic development policies, and so on. Since the reform and opening up for more than 40 years, the eastern coastal area has achieved rapid economic development due to its unique location advantages and national policies, and has formed an economic position with obvious comparative advantages. The impact of spillover effects has also gathered many advantageous resources from the central and western regions, and the industrial structure has gradually improved, forming a highly optimized “three, two, and one” industrial structure, which has been labor-intensive in the early stage. Resource dependence is strong, and the extensive economic growth mode based on factor input is gradually transformed into a connotative economic growth mode based on the tertiary industry and technological progress as the core, finally creating a green total factor productivity level in the eastern region. Significantly higher than the central and western regions and the national average, the central region has the strategic support of the country’s “Rise of the Central Region”, and the region has comparative advantages in human and natural resources, plus the central region’s status as a transportation hub and an important base for industrial raw materials. After the relevant industries in the coastal areas become saturated, it can effectively undertake the transfer of industries in the eastern region and attract a large amount of FDI investment to develop its own secondary industry; although the economic development of the western region is relatively backward, there is an objective and factual gap with the eastern and central regions. However, because the country established a heavy chemical industrial system in the western region in the early stage, which is characterized by resource dependence, high energy consumption, and high pollution, coupled with the support of the western development strategy and the transfer of industries from the east to the central region, the industrial development level of the western region is also high. It has been greatly improved. However, generally speaking, the industries in the central and western regions as a whole show an industrial pattern of “two, three, one”, andthe secondary industry represented by industry tends to develop at the expense of environment. The green total factor productivity of the western region is significantly lower than that of the eastern region, which is dominated by high-tech tertiary industries.

## 3. The Measurement Method of Government Environmental Information Disclosure and Time and Space Differentiation

The establishment of coordinated international cooperation to jointly reduce greenhouse gas emissions requires countries to have full confidence in each other’s monitoring and emission reduction of greenhouse gases. There is the need to rely on government departments to disclose energy and environmental protection information to the public. In May 2008, the Chinese government took a crucial step in promoting the disclosure of information on environmental protection—the introduction of the Regulations on Disclosure of Information by Government Departments and the Measures for Disclosure of Information by the Ministry of Environmental Protection. This is the first time that environmental protection agencies at all levels are required to disclose pollution data. The Public and Environmental Research Center (IPE) and the Natural Resources Conservation Association (NRDC) jointly released the Pollution Information Transparency Index (PITI) index system, and in the first year of the country’s release of information disclosure regulations, it will provide important pollutants to key polluting cities across the country. Therefore, there is a need to carry out systematic PITI evaluation and publish the PITI index. This index has become an important basis for evaluating the degree of government environmental information disclosure. Since this index is a measure of the policy implementation of each city, in order to obtain the result of measuring the province, this article replaces it with the average weighted value of the cities covered by the province, and the weight is the proportion of the city’s GDP. The PITI index evaluation method has been adjusted during the evaluation. In order to make the data comparable before and after, this paper compares the previous year’s data with the current year’s maximum value of the PITI index for dimensionless processing. The required data comes from the previous year’s PITI index report.

**Figure 1:**
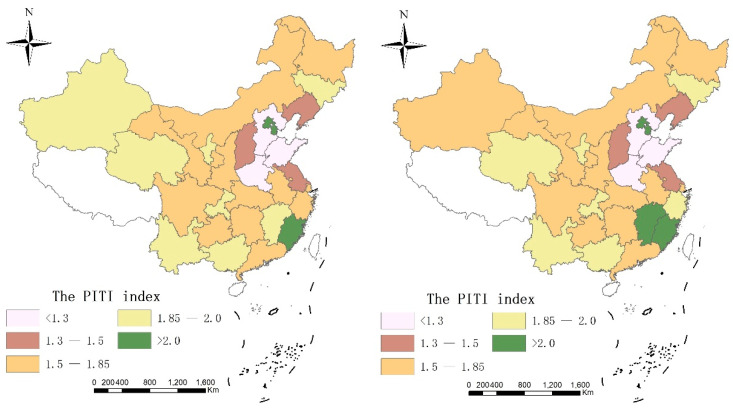
Spatio-temporal differentiation of China’s PITI index from 2010 to 2018.

In order to directly reflect the spatio-temporal characteristics of China’s PITI index during the period 2010–2018, this paper uses 2010 and 2018 as the time section and refers to the natural discontinuity classification method to draw the PITI index spatio-temporal differentiation map as shown in Figure 1.

Based on Figure 1, it can be seen that during the study period, China’s PITI index showed significant and stable spatial agglomeration characteristics, and since 2010, most of China’s provincial government environmental information disclosure has been at a relatively high level, including Beijing and Tianjin. The municipal and Fujian provincial governments have a high level of environmental information disclosure. The PITI indexes of related regions have long been in the first echelon of China, but the PITI indexes of Hebei, Shandong, and Henan provinces have always been relatively lagging behind, and there is a large gap with other provinces. The level of environmental information disclosure needs to be improved.

## 4. Data Selection and Model Construction

This article will conduct an empirical test, and the regression model is as follows:

(8) $${\mathrm{Gtfp}}_{\mathrm{it}}=\beta_{0}+\beta_{1}{*\mathrm{Piti}}_{\mathrm{it}}+\beta *Conrl_{\mathrm{it}}+\alpha +t$$

In the regression model of this article, Gtfp is the green total factor productivity of city *i* in year *t*, PITI is the government environmental information disclosure index, Conrl is the relevant control variable selected in this article, α is the unobservable fixed effect, and t is the time effect.

Explained variable: Green total factor productivity. According to the second part, we select the SBM function based on the slack directional distance proposed by Fukuyama and Weber, and the Luenberger productivity index with additive structure proposed by Chambers et al. to calculate the green total factor productivity, which is proposed in Oh [31]. Inspired by the idea of the global ML index, the SBM-based global directional distance function and the global Luenberger index are constructed to calculate the total factor productivity. The measurement method used in this paper avoids the phenomenon of “technological regression” and the possible non-solution of linear programming using mixed directional distance function, which makes the results transferable. It can not only analyze the short-term change of green total factor productivity, but also observe its long-term trend.

Core explanatory variables: According to the third part, the Pollution Information Transparency Index (PITI) is an important index to evaluate the degree of government environmental information disclosure; this paper selects the weighted average of the city-level PITI index as the indicator to measure government environmental information disclosure.

Control variables: Obviously, in addition to government environmental information disclosure, there are still other variables having an impact on the green total factor productivity. In order to control these impacts, this paper selects control variables from five dimensions: economic development, industrial structure, openness, employment attraction, and science and technology investment, as follows:

(1) **Economic development:** The improvement of economic development level will not only make the city accumulate more wealth and provide capital for realizing the long-term growth of urban economy, but also make people’s demand for a good environment more urgent, and per capita GDP is one of the core indicators reflecting the level of regional economic development [41,42]. Therefore, this paper selects per capita GDP as the control variable, and control the impact of economic development on green total factor productivity.
(2) **Industrial structure:** Industrial structure is an important factor affecting the urban ecological environment. It is generally believed that the waste gas, wastewater, and solid emissions formed in the process of industrial and agricultural production will cause great pollution to the environment [43,44], so the urban industrial structure will also affect the green total factor productivity. To control the impact of industrial structure on green total factor productivity, this paper selects the ratio of the secondary industry and the tertiary industry to GDP as the control variables of the dimension of industrial structure.
(3) **Openness:** The higher the level of urban opening to the outside world, the easier it is to introduce relatively advanced production technology and reduce environmental pollution. However, the improvement of urban openness may also lead to a large number of migrations of pollution intensive industries, resulting in a negative impact on the ecological environment [45]. Openness will further affect green total factor productivity by affecting environmental pollution. In order to control this impact, this paper takes the ratio of actually utilized foreign capital to GDP to measure the degree of regional openness.
(4) **Employment attraction:** On the one hand, employment attraction can measure the degree of regional aging, On the other hand, it can also explore the situation of urban human capital. The stronger employment attraction often means that it can attract more labor capital inflows, so that the total factor productivity has a more favorable capital base [46]. To control the impact of employment attraction, this paper measures employment attraction by the ratio of employment to the total population.
(5) **Scientific and technological investment:** According to the endogenous growth theory, scientific and technological innovation is the source of long-term economic growth. Scientific and technological innovation is an important way to improve economic efficiency and promote the growth of total factor productivity [47]. As the material basis of scientific and technological innovation, scientific and technological investment often promotes green innovation and has an impact on green total factor productivity. This paper takes the proportion of local government financial science and technology expenditure in GDP as the control variable to control the impact of science and technology investment on green total factor productivity.

Due to the availability of data, this chapter selects 2010–2018 as the research period. Specifically, the data sources used in this paper are as follows:

Explained variable: Green total factor productivity is a relative change. This chapter takes the GDP of each province from 2010 to 2018 as the expected output, wastewater and exhaust emissions as the unexpected output, and capital stock, employment, and total energy consumption as the input indicators to calculate the green total factor productivity from 2010 to 2018. The core explanatory variable PITI index comes from the Center for Public Environmental Research (IPE) and the Natural Resources Conservation Association (NRDC). The relevant data in the calculation of total factor productivity, such as wastewater and waste gas emissions, employment, total energy consumption and per capita GDP in the control variables, the proportion of the secondary industry, the proportion of the tertiary industry, the total amount of actually utilized foreign capital, employment, local government finance, and science and technology, are from China’s Urban Statistical Yearbook (2011–2019), China Environmental Statistics Yearbook (2011–2019), China Energy Statistics Yearbook (2011–2019), China Science and Technology Statistics Yearbook (2011–2019), and statistical yearbooks of provinces and cities in 2011–2019. In addition, the ratio of actually utilized foreign capital to GDP, the ratio of employed people to the total population, and the ratio of local financial science and technology to GDP are calculated on the basis of the above data, and the capital stock is calculated by the sustainable inventory method. The descriptive statistics of variables are shown in Table 2.

## 5. Regression Result

### 5.1. Baseline Regression

After Horsman’s test, this paper finally selects a two-way fixed-effect model. The Table 3 shows the benchmark regression results.

Therefore, it can be found that every increase of 1 in PITI will bring about an increase of about 0.2 units of green total factor productivity. Therefore, it is preliminarily judged that the increase in the level of government environmental information disclosure has a positive impact on green total factor productivity; in addition, it can be obtained. The following are the conclusions: (1) The increase in the proportion of the secondary industry and the proportion of the tertiary industry has a positive impact on green total factor productivity, but the proportion of the tertiary industry has a more significant impact on green total factor productivity. Therefore, industrialization and modernization service industrialization can promote the improvement of green total factor productivity, but relatively speaking, the development of modern service industry has a more obvious impact on green total factor productivity; (2) The actual use of foreign capital as a percentage of GDP and green total factor productivity are negative relations, therefore, while the positive effect of foreign capital utilization on China’s national economy is expanding, the introduction of foreign capital on many aspects of the host country’s economic impact, such as technology transfer and spillover effects, impact on the balance of payments and dependence issues. Potential issues such as industry monopoly, regardless of the current level of development, require us to study and think from a new perspective to avoid situations that endanger national economic security; (3) The proportion of the number of employees in the total number of people and the green total factor productivity are negative The reason is that cities with a relatively high number of employees are often large cities with influx of people. Therefore, we must be alert to the impact of metropolitan disease on green total factor productivity. Metropolitan disease refers to population expansion and traffic in large cities. “Symptoms” such as overcrowding, housing difficulties, environmental degradation, resource shortages, and high prices; (4) The proportion of government investment in technological innovation in GDP is negatively correlated with green total factor productivity. The reason may be that the government’s investment structure in technological innovation is not optimal.

After the aforementioned, the heteroscedasticity test was performed. Heteroscedasticity refers to the fact of the basic assumption that the variance of the random interference term is constant and is not established. It can be understood as the correlation with the sub-variables. This paper adopts the Breusch–Pagan test [48]. The test result is: chi2(1) is 0.65, and the *p*-value is 0.4194, so the assumption that there is no heteroscedasticity is acceptable.

On this aforementioned basis, the cross-sectional correlation test is performed. If there is correlation between the cross-sections of the panel data, the estimated coefficient will be biased. Therefore, this paper uses Pearson’s method to conduct the cross-sectional correlation test. The *p*-value is 0.1376, so it is acceptable that there is no cross-section. Assumptions of relevance are found in [49].

**Figure 2:**
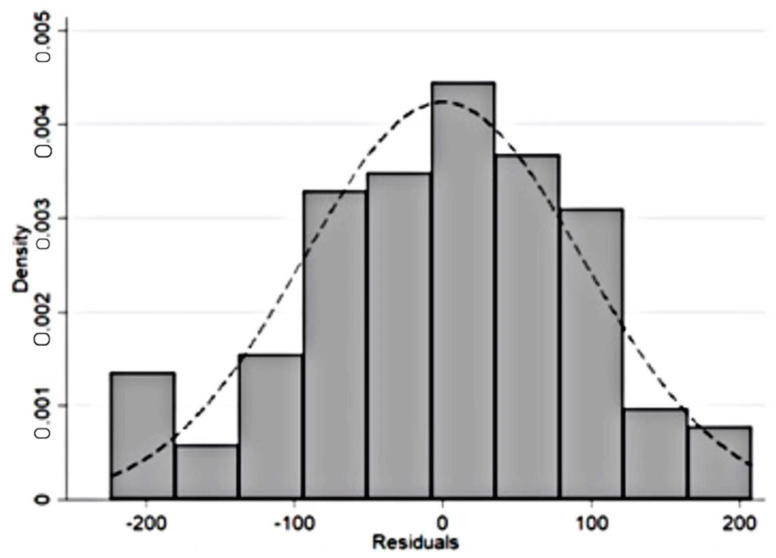
Residual distribution.

Finally, a normality test is performed. Normality testing refers to testing whether the basic assumption is that the residuals follow a normal distribution with a mean value of 0 holds. This paper uses Shapiro–Wilk to test the normality of the residuals [50], and the results are shown in Figure 2 and Table 4.After testing, the assumption that the residuals do not follow a normal distribution is rejected.

### 5.2. Robustness Test

The results of rubustness test are in Table 5. This article first uses the method of deleting extreme values to analyze the robustness test. The first and second columns are the results of deleting the top 5% and bottom 5% of the green total factor productivity respectively; the third and fourth columns are respectively the results of the top 5% and bottom 5% samples of the PITI index have been deleted. Later, considering that in high-level cities with a higher level of economic development and a greater degree of marketization, the government may have greater incentives to disclose environmental information. Therefore, the fifth column shows the results after deleting the top 10% of GDP. Therefore, it can be found that whether it is in any way of robustness testing, the positive promotion of green total factor productivity by government environmental information disclosure exists steadily.

### 5.3. Endogenous Analysis

Although this article has used the two-way fixed-effects model to deal with unobservable time effects and individual effects, there are still endogenous problems. Due to severe endogeneity, the least squares estimator will no longer be a consistent or optimal estimator. The general approach is to select appropriate instrumental variables to reduce the correlation between random items and endogenous explanatory variables. However, it is difficult to find suitable variables that are related to foreign technology spillovers and completely unrelated to total factor productivity. Therefore, this paper chooses the systematic GMM estimation method to solve the endogenous problem in the study of technology spillovers in import trade, the rusults are in Table 6.

The systematic GMM estimation method was originally proposed for estimating dynamic panels. It can use the level value and difference value of endogenous explanatory variables as instrumental variables to overcome the endogenous problem of explanatory variables without the need to seek other instrumental variables. Arellano and Bond [51] first proposed the first-order difference GMM estimation method, which can better solve the problem of biased and inconsistent estimates caused by endogenous explanatory variables. This estimation method first combines the original level equation and performs the difference, and then uses the level value of the endogenous explanatory variable lagging two orders and more than two orders as the instrumental variable of the endogenous explanatory variable difference item, because it is not related to the difference item of the random item, but is the difference with the endogenous explanatory variable item related. Rigobon [52] adopted the first-order difference GMM estimation method when solving the endogenous problem of international trade and economic growth. Although the first-order difference GMM estimation method can solve the endogeneity problem of explanatory variables very well, it may produce weak instrumental variables caused by insufficient instrumental variables. The basic idea is to solve the problem caused by weak instrument variables by adding new effective instrument variables. The specific method is to use the differential lag term of the endogenous explanatory variable as the level equation The instrumental variable of the endogenous explanatory variable, because it is related to the endogenous explanatory variable, but not related to the random item. Blundell and Bond [53] proved that the system GMM estimation method has better finite sample properties than the first-order difference GMM estimation method, and can reduce the bias caused by the first-order difference GMM estimation method to a large extent.

The Sargan test in the system GMM estimation is used to judge whether there are over-recognition constraints in the estimation process. The null hypothesis is that the selection of instrumental variables in the model is valid, and the Abanda (2) test is used to judge whether there are two differences in the residual term of the difference equation. In order serial correlation, the null hypothesis is that there is no serial correlation in the residual term of the difference equation. At the 5% significance level, the instrumental variables in the system GMM estimation are effective, and there is no serial correlation in the residual term of the difference equation. The results of the GMM estimation are shown in the following table. Therefore, the government environmental information disclosure and green total factor combine to be, overall, the positive matching relationship of factor productivity which still exists steadily.

## 6. Analysis of Heterogeneity Based on Panel Threshold Model

The impact of urban environmental information disclosure at different development stages on green total factor productivity may be different. According to modern economic growth theory, economic growth can be regarded as the process of an economy’s convergence from an initial state to a steady state, and this initial state. Almost all are pre-industrial societies with low capital per capita, while the steady state is an industrial society with high capital per capita. This has already implied the symbiosis of environmental pollution that cannot be avoided with economic development, because with the increase in per capita capital from low to high, from the pastoral era of reciprocity to the industrial era of roaring machines, not only the economic scale has occurred. Huge changes and profound changes have taken place in the industrial structure. Therefore, in cities at a lower development stage, the promotion of environmental information disclosure on green total factor productivity should be smaller than that of cities at a higher development stage with a panel threshold model for the threshold.

### 6.1. Threshold Eigenvalue Test

For a threshold model, the determination of the number of thresholds is necessary. We use the bootstrap method, which is the bootstrap method, to estimate the *p*-value by repeated sampling 500 times. The test results are shown in Table 7. The results show that the threshold model based on per capita GDP has single-threshold and double- threshold values. The *p*-value (0.000) of the single-threshold model test and the *p*-value (0.000) of the double-threshold model test are both at the significance level of 1% and 5%. These passed the test, but the three-threshold model test did not pass the significance test, so this paper uses single-threshold and double-threshold models for analysis [54].

### 6.2. Threshold Regression Results and Analysis

Therefore, this article establishes a single-threshold model:

(9) $${\mathrm{Gtfp}}_{it}=\lambda_{0}+\lambda_{1}D_{it}\cdot I(Gdpper_{it}\leq r_{1})+\lambda_{2}D_{it}\cdot I(Gdpper_{it}>r_{1})+\lambda_{0t}X_{it}+\gamma \cdot t+\varepsilon_{it}$$

Among them, *I*(·) represents the indicator function. When the expression in the brackets is true, the function value is 1, and when it is false, the function value is 0. *D_it_* is the core explanatory variable, *Gdpper_it_* is the threshold variable, *X_it_* is the control variable, and *ε* is the random disturbance. When *Gdpper_it_* ≤ *r*_1_, the *D_it_* coefficient is *λ*_1_; when *Gdpper_it_* > *r*_1_, the *D_it_* coefficient is *λ*_2_. *X_it_* is a variable other than the core explanatory variable, *t* is the time effect, and *λ*_0_ is a constant. We are concerned about the similarities and differences between *λ*_1_ and *λ*_2_.

The above model is suitable for the single-threshold case, the following model is suitable for the double-threshold case:

(10) $${\mathrm{Gtfp}}_{it}=\lambda_{0}+\lambda_{1}d_{it}\cdot I(Gdpper_{it}\leq r_{1})+\lambda_{2}d_{it}\cdot I(r_{1}<Gdpper_{it}<r_{2})+\lambda_{3}d_{it}\cdot I(r_{2}\leq Gdpper_{it})+\lambda_{0t}X_{it}+\gamma \cdot t+\varepsilon_{it}$$

According to the rusults in Table 8, it can be found that the higher the per capita GDP of the city, the greater the effect of government environmental information disclosure on the improvement of green total factor productivity; the reason is that in the initial stage of economic development, the marginal benefit of factor input is greater, and in the later stage of economic development, the marginal benefit of factor input is reduced, and high-quality development such as green development is more needed. High-quality development is also called high-quality economic development, and real economic development is high-quality development. High-quality economic development is a growth method with accurate economic data, optimized business environment, product quality assurance, accurate resource docking, and optimized allocation. It is an innovation-driven economic growth method and an innovative, high-efficiency, energy-saving, environmentally-friendly, and high-value-added growth method. Smart economy is the leading factor, high value-added core is the core, quality is leading the quantity, GDP has no moisture, making the economic aggregate an effective economic aggregate, promoting the continuous upgrading of industries, and promoting economic construction, political construction, cultural construction, social construction, and ecological civilization construction in a five-in-one, comprehensive, and sustainable growth mode.

## 7. Conclusions and Policy Implications

Government environmental information disclosure is a basic and overall work in the environmental governance system. Therefore, can government environmental information disclosure really improve green total factor productivity? If so, is there structural mutation and heterogeneity in the promotion effect of government environmental information disclosure on green total factor productivity? In order to answer the above questions, this paper calculates the green total factor productivity of Chinese provinces and cities based on the data of Chinese provinces and cities from 2010 to 2018. Then, based on the two-way fixed effect model, this paper finds that government environmental information disclosure can significantly promote the improvement of green total factor productivity. When the degree of government environmental information disclosure increases by one unit, and green total factor productivity will increase by 0.2 units. In order to make the results more accurate, this paper adopts systematic GMM estimation to deal with the endogenous problem. The results show that when the endogenous problem is considered, the government environmental information disclosure not only still promotes the green total factor productivity, but also enhances this effect. Furthermore, the results of threshold model show that the promotion effect of government environmental information disclosure on green total factor productivity has regional heterogeneity. When the regional economic level is relatively developed, the effect of government environmental information disclosure on green total factor productivity is relatively stronger.

According to the above conclusions, we recommend the following suggestions for relevant government departments to implement environmental information disclosure and ensure the sustainability of urban economic growth:

First, environmental information disclosure is not only a concrete embodiment of the innovative working mechanism and environmental supervision means of environmental protection departments, but also the objective need for environmental protection to actively adapt to the new normal of economic and social development. Environmental information disclosure can promote the improvement of green total factor productivity. Government departments can not only provide channels for the public to understand the dynamics of environmental protection and environmental information, but also promote the sustainable development of urban economic growth.

Second, local governments should formulate specific policies according to their regional characteristics when promoting environmental information disclosure and developing the sustainability of economic growth. For example, in the process of economic development, the regions with relatively advanced economic level should avoid realizing the rapid growth of economic aggregate at the cost of environment, and should increase investment in environmental pollution control measures such as environmental information disclosure, so as to realize the growth of economic quality. The areas with relatively backward economic level should improve the use efficiency of resources, pay attention to the cost performance of input and output, invest appropriately in pollution control measures such as environmental information disclosure, and actively improve the ecological environment with relatively limited resources, so as to develop the sustainability of economic growth.

Third, strengthen the capacity-building of environmental information disclosure of local governments and promote the active participation of all sectors of society. Environmental information disclosure is one of the effective ways to improve the ecological environment. Local governments should fully understand its practical significance in improving the ecological environment and realizing the sustainability of long-term economic growth, and actively strengthen the basic capacity-building of environmental information disclosure. In the meanwhile, the government should guide all sectors of society and the public to actively participate in environmental information disclosure, evaluate the environmental information disclosure implemented by the government, regularly carry out relevant summary work according to the evaluation effect, and improve the deficiencies in the work, to improve the efficiency of environmental information disclosure.

This paper makes an empirical study on the impact of government environmental information disclosure on green total factor productivity, which supplements the blank of relevant research, but there are still some deficiencies in this paper. For example, the Pollution Information Transparency Index used to measure government environmental information disclosure only covers a part of the cities in China. With the limitation of data, the average weighted value of the cities covered by the corresponding provinces is used to obtain the Pollution Information Transparency Index of each province and city in this paper. The research is carried out at the provincial and municipal level. In the future, when the data popularity increases, the research from the urban level may make the results more accurate. In the meanwhile, there are few indicators to measure the degree of government environmental information disclosure. This paper uses the Pollution Information Transparency Index (PITI) to measure the degree of government environmental information disclosure, but its evaluation system still has some limitations. How to measure the degree of government information disclosure more accurately is also an important problem worthy of discussion in future research.

## Figures and Tables

**Table 1:** Green total factor productivity calculation results in various regions during 2010–2018.

Year	The Whole Country	Eastern Region	Central Region	Western Region
2010	0.5980	0.6735	0.5312	0.5712
2011	0.6153	0.7108	0.5390	0.5752
2012	0.7013	0.8649	0.5828	0.6238
2013	0.5697	0.6542	0.5099	0.5287
2014	0.5800	0.6766	0.5153	0.5305
2015	0.6142	0.7412	0.5350	0.5446
2016	0.6306	0.7838	0.5405	0.5430
2017	0.6454	0.8279	0.5420	0.5380
2018	0.6712	0.8806	0.5525	0.5481

**Table 2:** The descriptive statistics of sample variables.

Types	Variables	Definition	Observations	Mean	Std. Dev.	Min	Max	Unit
Explained variable	Gtfp	The green total factor productivity	1059	1.0	0.1	0.2	1.6	–
Explanatory variable	PITI	The Pollution Information Transparency Index	1059	44.9	16.6	8.3	85.3	–
Control variable	Pgdp	The per capita GDP	1059	67,407.8	36,482.1	14,707.0	256,877.0	yuan/person
	Ssr	The ratio of second industry output to GDP	1059	49.5	10.1	15.7	89.8	%
	Tsr	The ratio of tertiary industry output to GDP	1059	43.3	11.2	9.8	81.0	%
	Or	The ratio of total actually utilized foreign capital to GDP	1059	0.4	0.5	0.0	8.6	%
	Tr	The ratio of employed persons to total population	1059	18.0	16.5	0.1	147.3	%
	Tpr	The ratio of local public expenditure for science and technology to GDP	1059	0.4	0.6	0.0	4.5	%

**Table 3:** Baseline regression results.

	(1)	(2)	(3)	(4)	(5)	(6)	(7)
PITI	0.231 *** (10.12)	0.204 *** (10.18)	0.258 *** (8.11)	0.240 *** (7.47)	0.223 *** (7.36)	0.212 *** (7.66)	0.211 *** (7.62)
Gdpper		−1.2 × 10^−8^ *** (−2.81)	−1.2 × 10^−8^ *** (−2.85)	−1.3 × 10^−8^ *** (−2.97)	−1.3 × 10^−8^ *** (−3.06)	−1.2 × 10^−9^ *** (−3.06)	−3.6 × 10^−9^ (−0.83)
Ssr			−0.001 ** (−2.21)	−0.002 (−0.192)	−0.002 (1.71)	0.002 (1.44)	0.002 * (1.88)
Tsr				0.003 ** (2.34)	0.003 ** (2.87)	0.003 ** (2.95)	0.004 *** (3.18)
Or					−1.601 *** (−2.87)	−1.357 *** (−2.66)	−1.066 ** (−1.98)
Tr						−3.2 × 10^−6^ *** (−9.68)	−2.1 × 10^−6^ *** (−4.82)
Tpr							−3.516 *** (−3.98)
Time effect	control	control	control	control	control	control	control
Individual effect	control	control	control	control	control	control	control
Constant	0.522 *** (59.19)	0.523 *** (59.28)	0.590 *** (18.77)	0.330 *** (2.85)	0.283 ** (2.46)	0.330 *** (2.99)	0.285 ** (2.59)
R^2^	0.4993	0.5057	0.5104	0.5156	0.5295	0.6042	0.6181

Note: ***, **, * indicate significance at the significance level of 1%, 5%, and 10%, respectively.

**Table 4:** Shapiro–Wilk W test result.

Variables	Observations	W	V	Z	*p* > z
Residual	279	0.99	1.17	0.35	0.37

**Table 5:** Robustness test results.

	(1)	(2)	(3)	(4)	(5)
PITI	0.177 *** (7.64)	0.206 *** (6.96)	0.184 *** (6.72)	0.213 *** (7.08)	0.199 *** (7.39)
Gdpper	−2.7 × 10^−9^ *** (−0.82)	−3 × 10^−9^ (−3.85)	−3 × 10^−9^ (−0.74)	−3.7 × 10^−9^ (3.98)	−7.61 × 10^−9^ (−1.19)
Ssr	0.001 (1.21)	0.002 (1.24)	0.003 ** (2.07)	0.003 * (1.85)	0.003 *** (2.88)
Tsr	0.002 ** (2.15)	0.004 *** (2.99)	0.004 *** (3.21)	0.004 *** (3.28)	0.004 *** (3.61)
Or	−0.391 (−0.90)	−1.41 ** (−2.45)	−1.03 * (−1.96)	−2.281 *** (−2.78)	−0.728 (−1.46)
Tr	−7 × 10^−7^ * (−1.68)	−2.1 × 10^−6^ *** (−4.72)	−2 × 10^−6^ *** (−5.26)	−2.1 × 10^−6^ *** (−4.38)	−2 × 10^−6^ *** (−4.52)
Tpr	−1.961 ** (−2.45)	−3.437 *** (−3.81)	−3.671 *** (−4.18)	−3.234 *** (−3.53)	−3.821 *** (−4.45)
Time effect	control	control	control	control	control
Individual effect	control	control	control	control	control
Constant	0.398 *** (4.50)	0.340 *** (2.98)	0.280 ** (2.60)	0.278 *** (2.44)	0.217 ** (2.14)
R^2^	0.5288	0.739	0.622	0.6186	0.6243

Note: ***, **, * indicate significance at the significance level of 1%, 5%, and 10%, respectively.

**Table 6:** GMM regression results.

	(1)	(2)	(3)	(4)	(5)	(6)	(7)
PITI	0.005 *** (4.84)	0.005 *** (4.99)	0.004 *** (4.17)	0.003 *** (3.03)	0.003 *** (3.01)	0.003 *** (3.10)	0.003 *** (3.16)
Gdpper		−1.6 × 10^−8^ *** (−3.38)	−1.6 × 10^−8^ *** (−3.38)	−1.6 × 10^−8^ *** (−3.51)	−1.7 × 10^−8^ *** (−3.56)	−8.2 × 10^−9^ * (−1.71)	−8.1 × 10^−9^ * (−1.68)
Ssr			−0.001 ** (−2.08)	0.003 ** (2.40)	0.003 *** (2.80)	0.003 *** (2.73)	0.003 *** (2.70)
Tsr				0.004 ** (3.63)	0.005 ** (4.17)	0.005 ** (4.07)	0.005 ** (3.98)
Or					−2.585 *** (−2.81)	−2.319 *** (−2.57)	−2.381 *** (−5.57)
Tr						−2.1 × 10^−6^ *** (−5.57)	−2.1 × 10^−6^ *** (−4.21)
Tpr							−3.516 *** (−3.98)
Time effect	control	control	control	control	control	control	control
Individual effect	control	control	control	control	control	control	control
Constant	0.393 *** (8.17)	0.387 *** (8.07)	0.4750 *** (7.50)	0.126 *** (2.85)	0.078 (0.68)	0.096 *** (0.68)	0.093 ** (0.81)
R^2^	0.4104	0.4285	0.4438	0.4415	0.4568	0.5218	0.5218

Note: ***, **, * indicate significance at the significance level of 1%, 5% and 10%, respectively.

**Table 7:** Test results of threshold eigenvalues.

Model	F-Value	*p*-Value	Critical Value		
			1%	5%	10%
Single-threshold model	68.759 ***	0.000	15.805	10.391	7.450
Double-threshold model	42.669 ***	0.000	−2.596	−6.804	−10.843
Three-threshold model	−45.833	0.677	−14.342	−18.654	−22.558

Note: ***, **, * indicate significance at the significance level of 1%, 5%, and 10%, respectively.

**Table 8:** Regression results of panel threshold model.

Model	Variable	Value Range of GDP per Capita	Coefficient	95% Confidence Interval	
Single-threshold model	PITI	GDP per < 107,555)	0.192 *** (7.39)	0.0011075	0.0019089
		GDP per ≥ 107,555	0.307 *** (10.82)	0.0021626	0.0031205
Single-threshold model		GDP per < 75,563	0.188 *** (7.39)	0.0014725	0.0025375
		75563 ≤ GDP per <114,746	0.323 *** (11.62)	0.0025801	0.0036286
		GDP per ≥ 114,746	0.504 *** (14.49)	0.0039648	0.005207

Note: ***, **, * indicate significance at the significance level of 1%, 5%, and 10%, respectively.

## References

[1] Liu S., Fan F., Zhang J.Q. (2019). Are Small Cities More Environmentally Friendly? An Empirical Study from China. Int. J. Environ. Res. Public Health.

[2] Ke H.Q., Dai S.Z. (2021). Does Innovation Efficiency Inhibit the Ecological Footprint? An Empirical Study of China’s Provincial Regions. https://www.tandfonline.com/doi/abs/10.1080/09537325.2021.1959910.

[3] Zhu Q. (2005). The absence and remedy of environmental right to know—From kaixian Blowout accident. Law Sci..

[4] Wang J.S., Zhang B. (2019). Quality of environmental information disclosure and enterprise characteristics Based on heavily polluted industries of A-share in the Shanghai Stock Exchange. Manag. Environ. Qual..

[5] Yu Z., Jian J., He P. (2011). The Study on the Correlation between Environmental Information Disclosure and Economic Performance-With empirical data from the manufacturing industries at Shanghai Stock Exchange in China. Energy Procedia.

[6] Shi B., Feng C., Kang R. (2019). Environment Information Announcement and Structure Optimization of FDI. China Ind. Econ..

[7] Evans M.F., Gilpatric S.M., Liu L. (2009). Regulation with Direct Benefits of Information Disclosure and Imperfect Monitoring. J. Environ. Econ. Manag..

[8] Bennear L.S., Olmstead S.M. (2008). The impacts of the ‘right to know’: Information disclosure and the violation of drinking water standards. J. Environ. Econ. Manag..

[9] Cohen M.A., Santhakumar V. (2007). Information disclosure as environmental regulation: A theoretical analysis. Environ. Resour. Econ..

[10] Lu M., Feng H. (2014). Agglomeration and emission reduction: An empirical study on the impact of city size disparities on industrial pollution intensity. J. World Econ..

[11] Canfei H., Huang Z., Xinyue Y. (2014). Spatial Heterogeneity of Economic Development and Industrial Pollution in Urban China. Stoch. Environ. Res. Risk Assess..

[12] Copeland B.R., Taylor M.S. (2004). Trade, Growth, and the Environment. J. Econ. Lit..

[13] Huang M., Lin S. (2013). Pollution Damage, Environmental Management and Sustainable Economic Growth-Based on the Analysis of Five-Department Endogenous Growth Model. Econ. Res. J..

[14] Ke H.Q., Dai S.Z., Yu H.C. (2021). Spatial effect of innovation efficiency on ecological footprint: City-level empirical evidence from China. Environ. Technol. Innov..

[15] Fan F., Lian H., Liu X. (2020). Can environmental regulation promote urban green innovation Efficiency? An empirical study based on Chinese cities. J. Clean. Prod..

[16] Wang S., Jia M., Zhou Y. (2019). Impacts of changing urban form on ecological efficiency in China: A comparison between urban agglomerations and administrative areas. J. Environ. Plan. Manag..

[17] Bank W. (2016). ‘Green’ Growth, ‘Green’ Jobs and Labor Markets. https://openknowledge.worldbank.org/handle/10986/3277.

[18] Wang X., Chunyou W.U., Wensong Y.U. (2015). International Comparison on Determinants of Green Growth—Empirical Analysis on Panel Data of G20. http://en.cnki.com.cn/Article_en/CJFDTotal-BLDS201506002.htm.

[19] Elsadig M.A. (2012). Green TFP Intensity Impact on Sustainable East Asian Productivity Growth—ScienceDirect. Econ. Anal. Policy.

[20] Zhang Q., Yan F., Li K. (2019). Impact of market misallocations on green TFP: Evidence from countries along the Belt and Road. Environ. Sci. Pollut. Res..

[21] Xin B.G., Qu Y.M. (2019). Effects of Smart City Policies on Green Total Factor Productivity: Evidence from a Quasi-Natural Experiment in China. Int. J. Environ. Res. Public Health.

[22] Zhao M., Liu F., Sun W. (2020). The Relationship between Environmental Regulation and Green Total Factor Productivity in China: An Empirical Study Based on the Panel Data of 177 Cities. Int. J. Environ. Res. Public Health.

[23] Li X.M., Shi P.F., Han Y.Z. (2020). Measurement and Spatial Variation of Green Total Factor Productivity of the Tourism Industry in China. Int. J. Environ. Res. Public Health.

[24] Wang X.L., Wang L., Wang S. (2021). Marketisation as a channel of international technology diffusion and green total factor productivity: Research on the spillover effect from China’s first-tier cities. Technol. Anal. Strateg. Manag..

[25] Yu H.C., Zhang J.Q., Zhang M.Q. (2021). Cross-National Knowledge Transfer, Absorptive Capacity, and Total Factor Productivity: The Intermediary Effect Test of International Technology Spillover. https://www.semanticscholar.org/paper/Cross-national-knowledge-transfer%2C-absorptive-and-Yu-Zhang/1a9463c90f4af15475c796610bcb0417c46b4d69.

[26] Bradford D.F., Oates W.E. (1971). The Analysis of Revenue Sharing in a New Approach to Collective Fiscal Decisions. Q. J. Econ..

[27] Wang H.W., Cai L., Zeng W. (2011). Research on the Evolutionary Game of Environmental Pollution in System Dynamics Model. J. Exp. Theor. Artif. Intell..

[28] Zheng S., Wan G., Sun W., Luo D. (2013). Public demand and urban environmental governance. Manag. World.

[29] Fukuyama H., Weber W.L. (2009). Estimating indirect allocative inefficiency and productivity change. J. Oper. Res. Soc..

[30] Chambers R.G., Färe R. (1996). Productivity growth in APEC Country. Pac. Econ. Rev..

[31] Oh D.H. (2010). A global Malmquist-Luenberger productivity index. J. Product. Anal..

[32] Fan F., Du D.B. (2014). The Measure and the Characteristics of Temporal-spatial Evolution of China Science and Technology Resource Allocation Efficiency. J. Geogr. Sci..

[33] Fan F., Lian H., Wang S. (2020). Can regional collaborative innovation improve innovation efficiency? An empirical study of Chinese cities. Growth Change.

[34] Wang S., Wang J., Wei C. (2021). Collaborative innovation efficiency: From within cities to between cities—Empirical analysis based on innovative cities in China. Growth Change.

[35] Sun C.Z., Yan X.D., Zhao L.S. (2021). Coupling Efficiency Measurement and Spatial Correlation Characteristic of Water-Energy-Food Nexus in China. https://www.sciencedirect.com/science/article/abs/pii/S0921344920304687.

[36] Wang S., Wang X.L., Lu F. (2020). The impact of collaborative innovation on ecological efficiency—Empirical research based on China’s regions. Technol. Anal. Strateg. Manag..

[37] Wang S., Zhang J.Q. (2019). The symbiosis of scientific and technological innovation efficiency and economic efficiency in China—An analysis based on data envelopment analysis and logistic model. Technol. Anal. Strateg. Manag..

[38] Zhang J.Q., Chen T.T. (2018). Empirical Research on Time-Varying Characteristics and Efficiency of the Chinese Economy and Monetary Policy: Evidence from the MI-TVP-VAR Model. Appl. Econ..

[39] Chung Y.H., Färe R., Grosskopf S. (1997). Productivity and Undesirable Outputs: A Directional Distance Function Approach. Microeconomics.

[40] Boussemart J.P., Briec W., Kerstens K. (2003). Luenberger and Malmquist Productivity Indices: Theoretical Comparisons and Empirical Illustration. Bull. Econ. Res..

[41] Yu H.C., Liu Y., Liu C.L. (2018). Spatiotemporal Variation and Inequality in China’s Economic Resilience across Cities and Urban Agglomerations. Sustainability.

[42] Fan F., Zhang X.R., Yang W.Y., Liu C.L. (2021). Spatiotemporal Evolution of China’s ports in the International Container Transport Network under Upgraded Industrial Structure. Transp. J..

[43] Wang Z., Zong Y., Dan Y., Jian S.J. (2021). Country risk and international trade: Evidence from the China-B & R countries. Appl. Econ. Lett..

[44] Xiao Z.L., Du X.Y. (2017). Convergence in China’s high-tech industry development performance: A spatial panel model. Appl. Econ..

[45] Zhu Q.Y., Sun C.Z., Zhao L.S. (2021). Effect of the marine system on the pressure of the food–energy–water nexus in the coastal regions of China. J. Clean. Prod..

[46] Fan F., Zhang X.R. (2021). Transformation effect of resource-based cities based on PSM-DID model: An empirical analysis from China. Environ. Impact Assess. Rev..

[47] Fan F., Zhang K.K., Dai S.Z. (2021). Decoupling Analysis and Rebound Effect between China’s Urban Innovation Capability and Resource Consumption. https://www.tandfonline.com/doi/abs/10.1080/09537325.2021.1979204.

[48] Jing W., Zhang L. (2014). Environmental Regulation, Economic Opening and China’s Industrial Green Technology Progress. Econ. Res. J..

[49] Fan F., Dai S.Z., Zhang K.K. (2021). Innovation agglomeration and urban hierarchy: Evidence from Chinese cities. Appl. Econ..

[50] Pesaran M.H. (2004). General diagnostic tests for cross section dependence in panels. Camb. Work. Pap. Econ..

[51] Arellano M., Bond S. (1991). Some Tests of Specification for Panel Data: Monte-Carlo Evidence and an Application to Employment Equations. Rev. Econ. Stud..

[52] Rigobon R. (2003). Identification Through Heteroskedasticity. Rev. Econ. Stat..

[53] Blundell R., Bond S. (1998). Initial conditions and moment restrictions in dynamic panel data models. J. Econom..

[54] Liu N., Fan F. (2020). Threshold effect of international technology spillovers on China’s regional economic growth. Technol. Anal. Strateg. Manag..

